# The graduation shift of German universities of applied sciences

**DOI:** 10.1371/journal.pone.0210160

**Published:** 2019-01-25

**Authors:** Lutz Bornmann, Klaus Wohlrabe, Sabine Gralka

**Affiliations:** 1 Administrative Headquarters of the Max Planck Society, Division for Science and Innovation Studies, Munich, Germany; 2 ifo Institute, Munich, Germany; 3 TU Dresden, Dresden, Germany; Universidade de Mogi das Cruzes, BRAZIL

## Abstract

In research into higher education, the evaluation of completion and dropout rates has generated a steady stream of interest for decades. While most studies only calculate quotes using student and graduate numbers for both phenomena, we propose to additionally consider the budget available to universities. We transfer the idea of the excellence shift indicator [1] from the research to the teaching area, in particular to the completion rate of educational entities. The graduation shift shows the institutions’ ability to produce graduates as measured against their basic academic teaching efficiency. It is an important advantage of the graduation shift that it avoids the well-known heterogeneity problem in efficiency measurements. Our study is based on German universities of applied science. Given their politically determined focus on education, this dataset is well-suited for introducing and evaluating the graduation shift. Using a comprehensive dataset covering the years 2008 to 2013, we show that the graduation shift produces results, which correlate closely with the results of the well-known graduation rate and standard Data Envelopment Analysis (DEA). Compared to the graduation rate, the graduation shift is preferable because it allows to take the budget of institutions into account. Compared to the DEA, the computation of the graduation shift is easy, the results are robust, and non-economists can understand them results. Thus, we recommend the graduation shift as an alternative method of efficiency measurement in the teaching area.

## Introduction

In times of new public management, universities are no longer solely interested in measures of research excellence, but also in the efficiency of research: Can the given input (in terms of employees or expenditures) efficiently be transformed into research output (in terms of publications or patents)? Bornmann et al. [1] introduced the excellence shift to assess the efficiency of (higher) education institutions in conducting (successful) research. The method makes it possible to avoid the well-known heterogeneity problem in efficiency research, with either the data or the institutions being too varied to be fairly compared [2, 3]. Institutions are heterogeneous for many reasons, but they differ primarily in their location and focus: institutions are located in varying states and operate thus under conditions that are not comparable. Some universities emphasize research whereas others are more teaching oriented. The advantage of the excellence shift is that institutions are compared based on their own basic efficiency, which avoids comparing disparateness. For the calculation, two output variables depicting the institutional research side are used, whereby one is the subset of the other. The term “shift” refers to the fact that the indicator measures not only the efficiency of institutions, but also the magnitude of change, if the basic efficiency level is compared to an excellence level. For the excellence shift, Bornmann et al. [1] employ the total number of papers and the number of highly-cited papers as a subset. Based on these data sources, the shift shows the institutions’ ability to produce highly-cited papers as measured against their basic academic research efficiency (using institutions’ total budget as input indicator).

This paper makes three contributions to the literature:

Firstly, we transfer the idea of the excellence shift from the research area [1] to the teaching area, particularly to the completion rate of educational entities. Completion and dropout are topics of consistently high interest in research on higher education [4, 5], especially in Germany [6]. We call the transferred approach *graduation shift*. It is based on two output variables: the number of students at a university, which signals how attractive a university is, and the number of graduates, indicating how successful the graduation process works. The number of graduates is a subset of the number of students who have enrolled at that university. The main input variable is the expenses of the institution. The graduation shift then shows the institution’s ability to produce graduates as measured against their basic academic teaching efficiency. The output and input variables used in this study are an established choice for efficiency studies, which have been used by Agasisti and Dal Bianco [7], for example. To compare the results of the graduation shift with the results of a standard efficiency method, we also employ the variables in a Data Envelopment Analysis (DEA). Additionally, we contrast the results of our graduation shift to a simple measure of completion: the graduation rate, which shows how many students graduate from the university.
Secondly, unlike the previous literature looking at conventional universities, we deliberately use teaching data for universities of applied sciences (so-called *Fachhochschulen*). The institutions complement the existing German conventional universities by having a politically predefined focus on education (and not research or research training). They emerged in the 1960s, in response to the need for skilled labor and the growing demand for student places. Graduates receive the same formal title, but differ from leavers of conventional universities through their place of study. Most of the institutions are multidisciplinary, vocationally oriented and align their subject range to suit the regional economy [8]. Hence, our teaching oriented efficiency approach is perfectly suited to assess their effectiveness. Despite their growing status within the German higher education sector, with half of all existing institutions being universities of applied sciences, they have only rarely been subject to efficiency studies to date.
Thirdly, we outline some drawbacks of the excellence shift. For example, it does not appropriately account for the input parameters in specific situations.

This paper starts with a brief overview of the efficiency literature as well as a description of our data set. Afterwards, the graduation shift approach is explained. In the final sections, we present the graduation shift results and compare them with the well-known graduation rates and the results from the DEA.

## Related literature

De Witte and López Torres [2] and Rhaiem [9] provide excellent summaries of the efficiency literature in the education sector. The term “efficiency” is defined as the success of maximizing the output from a given set of inputs (or vice versa). The efficiency of educational entities emerged as a topic of early interest, with initial studies recommending relevant input, as well as output variables [10] and later studies discussing limitations, especially in terms of the comparability of universities [11]. While the productivity of conventional universities has been frequently analyzed in the past (see exemplary [12, 13, 14] for evaluations of the German HE Sector and [15] for a cross-country comparison), only two studies have examined the efficiency of universities of applied sciences to date [16, 17]. Both studies classified the universities as just one component of the higher education sector and therefore examined them as part of a bigger sample. Olivares and Wetzel [16] thereby focus the analysis on the economies of institutional scale and scope. The authors applied a recent specification of the Stochastic Frontier Analysis (SFA) to an unbalanced panel, covering 72 conventional universities and 80 applied institutions during the time period from 2001 to 2008. Their results show that all entities work on a similarly high level of efficiency and exhibit increasing returns to scale. With a similarly mixed, but much smaller sample, Başkaya und Klumpp [17] used the DEA in a cross-section of 33 institutions. Their evaluation reveals that the universities of applied science exhibit heterogeneous efficiency scores on a low average level. The differences between the results of the studies by Olivares and Wetzel [16] and Başkaya und Klumpp [17] are primarily caused by the differences in the respective efficiency approaches and considered variables, for example, for the representation of the teaching output. Agasisti und Haelermans [18] illustrate how sensitive the efficiency values are to the variable representing the teaching output (number of students or graduates at each institution). A further valuable addition to the literature on universities of applied sciences are the publications by the German Council of Science and Humanities (Wissenschaftsrat), which give a thorough view of the universities of the applied science landscape (see, for example [19]). Although the council separately evaluated the input and output variables of the universities, it missed the opportunity to evaluate their efficiency.

Within the literature on efficiency of higher education institutions, the teaching side of universities is most commonly represented by the number of students or graduates. The majority of authors thereby considers the absolute number or split the overall amount according to different levels of education or subject groups. Variables reflecting the quality of education or representing the completion rate are rarely included. Notable exceptions are the following three studies: Agasisti [20] evaluated the efficiency of Italian institutions. The author distinguished between students (graduates), who finished their studies within the regular duration of the course, and the overall number of students. Whereas Zoghbi, Rocha and Mattos [21] considered dropout numbers in their study and Sav [22] took account of the student fall to fall semester return (retention) in their efficiency evaluations.

## Data and methods

The initial sample consists of 262 German public universities of applied science (classified by the Federal Statistical Office of Germany) including 163 private and/or specialized institutions (the latter are primarily located in theology, art, and pedagogy). These private and specialized institutions have not been considered in this study, mainly due to their different funding arrangements. Due to mergers of institution and missing data, 18 further institutions had to be dropped. The final sample thus comprises 81 of the 99 German public universities of applied science. To gain insights into the productivity of these institutions, we evaluated their primary activity, namely teaching, with respect to their main input, i.e. expenses. The output variable “teaching” is represented by the total number of first semester students alongside the graduates from bachelor and master courses (or equivalent). The Federal Statistical Office of Germany distinguishes between students in their first subject related semester (in German: *Fachsemester*) and their first university semester (in German: *Hochschulsemester*). We deliberately used students in the first *Fachsemester*, since it comprises students in their first *Hochschulsemester* and also envelopes students who changed their field of study. Student numbers refer to the academic years 2008/2009 through 2013/14 and financial variables are from 2008 to 2013. The data were provided by the Federal Statistical Office of Germany. Expenditure data are deflated to the year 2013. Table 1 reports descriptive statistics for the year 2013. The values are similar to those reported by Olivares and Wetzel [16]. An institution has around 1,400 students in the first semester and around 1,100 graduating in that year. Average expenditure amounts to 34 million euros per university. The largest institution among the 81 universities of applied science is the FH Cologne with respect to both students and expenditure.

**Table 1:** Descriptive statistics.

	Universities of Applied Science (n = 81)			
	Mean	Standard Deviation	Minimum	Maximum
First Semester Student (2008–10) ^a^	1,383	679	336	4,246
Graduates (2011–13)	1,074	546	238	3,277
Expenditures (2008–13) ^b^	34	20	5	142
Employees (2008–13)	377	203	79	1315

^a^ First semester students are defined as students within their first subject-related semester.

^b^ In € million, 2013 prices.

**Fig 1:**
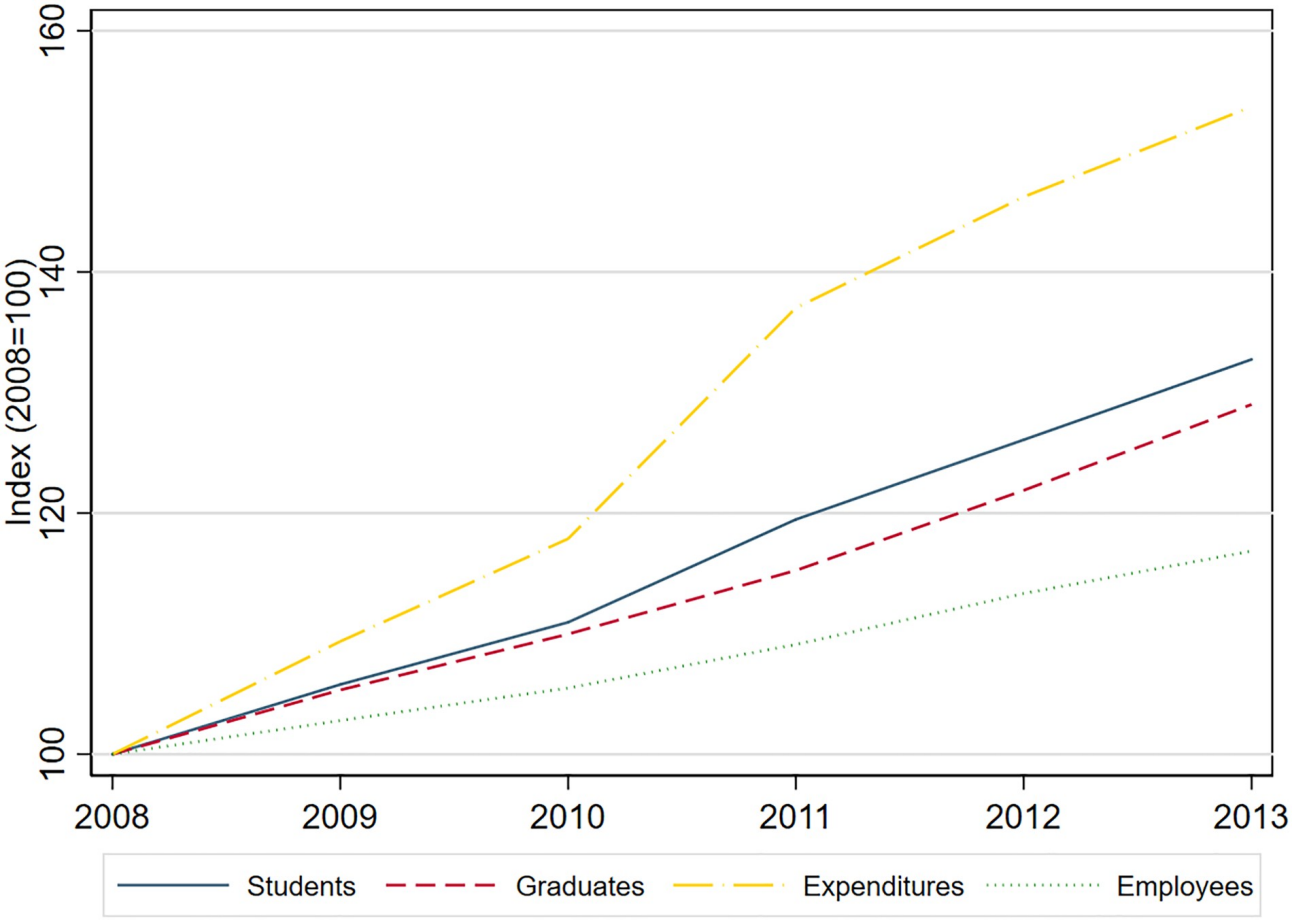
Development over time for inputs and outputs.

Fig 1 shows the change in average students, graduates, and expenses over the considered timeframe. While the first two variables show a moderate and similar increase, the expenditures grew to a larger extent.

A crucial point is the definition of the point in time when a student graduates. This is surely not the same for students from different universities. For a discussion of the problems in measuring time to degree in the German higher education sector see Theune [23]. Therefore, given our data framework, we have to make some assumptions, based on the standard and actual duration of study. While bachelor (master) students in Germany have a standard period of study with 6 (4) semesters, the actual period of study is listed as 7.3 (4.2) semesters by the Federal Statistical Office of Germany [24]. Since we have a mixed sample, featuring both bachelor and master students, we assume an average study duration of 6 semesters. Hence, we split the overall sample (with a period from 2008 to 2013) according to the contained student cohorts and obtain three groups. Based on our assumption of six semesters, the first semester students from 2008 (2009 or 2010, respectively) have been related to the graduates of 2011 (2012 or 2013, respectively). For each cohort, the average expenditure over the corresponding three years has been calculated.

### The graduation shift

We have one input and two output variables. On the output side, our approach is based on two indicators: (1) total number of first semester students (*S*) and (2) total number of graduates (*G*). The input is defined as the total expenditure (*E*).

Given our dataset, the graduation shift is formally calculated as follows [1]:

1. The relative shares *p*_1*i*_ = *S*_*i*_/∑*S*_*i*_; *p*_2*i*_ = *G*_*i*_/∑*G*_*i*_ and *ex*_*i*_ = *E*_*i*_/∑*E*_*i*_ are calculated. These represent the share of each university given the sum of inputs and outputs. The percentages standardise the absolute numbers and make them comparable across indicators.
2. The university efficiency scores for the two outputs given by *e*_1*i*_ = *p*_1*i*_/*ex*_*i*_ and *e*_2*i*_ = *p*_2*i*_/*ex*_*i*_ are calculated. These are simple productivity measures relating the outputs to the input.
3. The difference of the two efficiency scores *e*_2_ –*e*_1_ defines the graduation shift. The score can be interpreted only in relative terms.

The term “shift” points to the direction and magnitude of change in productivity of a university compared to its basic efficiency. The framework of the graduation shift allows considering one input and two outputs simultaneously. Thereby one output has to be a subset of the other output indicator.

To gain an impression of the graduation shift’s robustness with respect to outliers, we experimented with extreme values from our data set. These analyses show that the index changes only marginally and the resulting rankings remained almost unchanged.

### The DEA

To relate the results from the graduation shift to the results of an established method, we additionally performed an efficiency analysis as a benchmark. Two main methods for estimating efficiency coexist for the educational sector. In both cases, inefficiency is measured by the distance of each institution to a calculated efficiency frontier. Since the frontier is determined by the sample, efficiency is a relative measure: the efficiency of a particular institution is calculated relative to the performance of the other institutions in the sample.

We choose the non-parametric DEA introduced by Charnes, Cooper and Rhodes [25] as a benchmark for this study, because it is the most frequently used method and it can be implemented in a straight-forward manner. Using linear programming, the frontier and the position of each entity are calculated by the ratio of (weighted) outputs over (weighted) inputs. Detailed overviews of advantages and variations of the DEA can be found in Bogetoft and Otto [26] as well as Wilson and Clemson [27]. To achieve the best possible benchmark, we performed the DEA with the same dataset as used for the graduation shift, considering first semester students and expenditure as inputs and graduates as output. We allow for Variable Returns to Scale (VRS) and choose the output-oriented approach, assuming that universities maximise their output with the given input.

The DEA has the advantage that it can handle various numbers of inputs and outputs simultaneously. In principle, one could consider the number of employees or the physical capital (as the number of computers or laboratories) as inputs, alongside the expenditures. Since this is not possible with the graduation shift, we included in the DEA the same restricted number of inputs and outputs as in the graduation shift calculation. To test the robustness of the results, we additionally vary the considered input, given by the expenditures in our baseline model, and consider the number of employees instead. One potential drawback of the DEA is that the results can be sensitive to outliers (see Gnewuch and Wohlrabe [28]). Since the graduation shift is not sensitive to outliers, the new approach has this advantage over the DEA.

## Results

**Fig 2:**
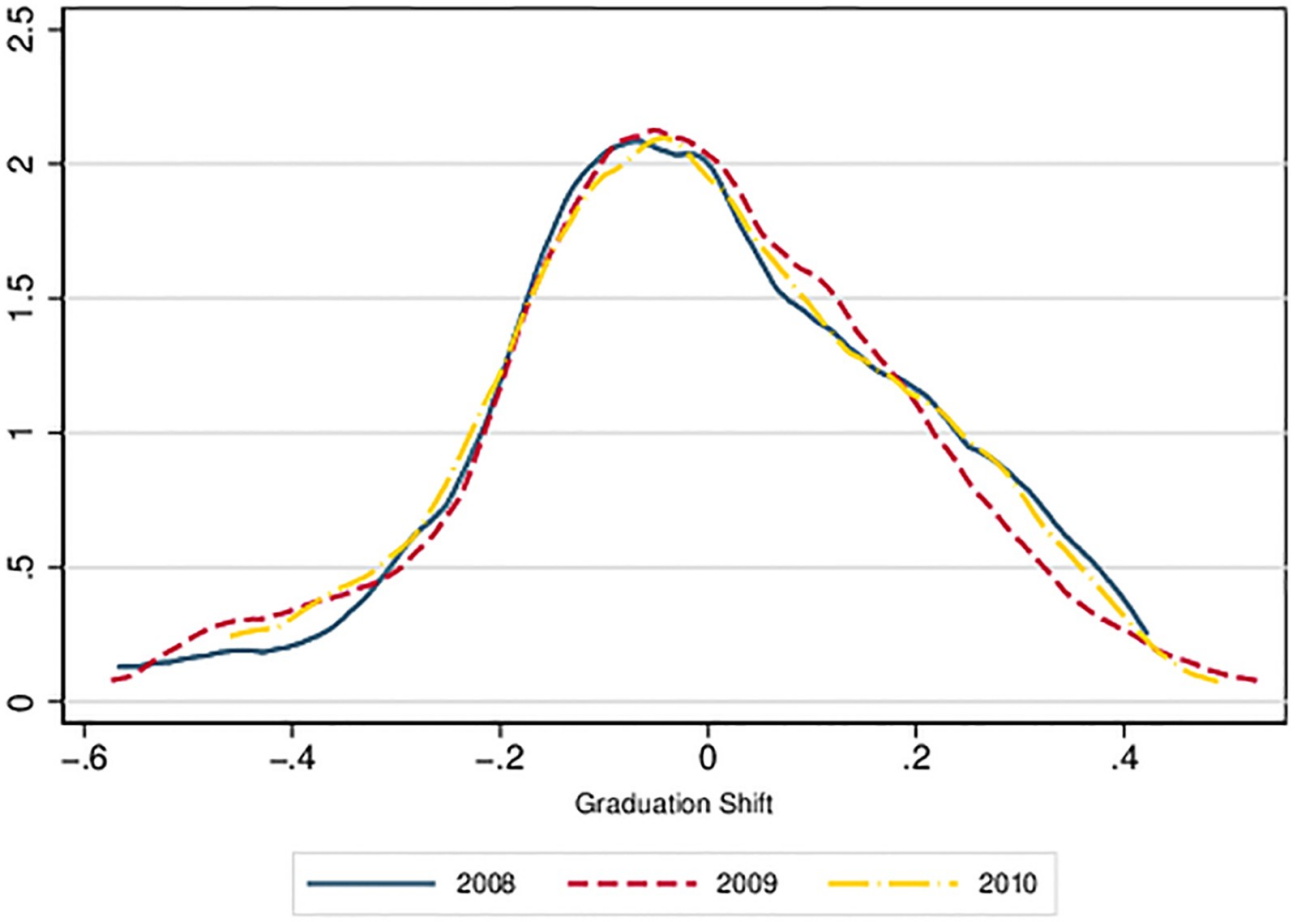
Kernel estimates of the graduation shift (2008–2010).

In the first step, we calculated the graduation shift for each year from 2008 to 2010. Fig 2 plots the corresponding kernel estimate of all 81 scores for every cohort year. It shows that the distribution is constant across time. Both mean and median are negative. There are more negative than positive scores on average over the three years. However, a visual inspection of the results reveals that the relative positions of the universities are volatile with respect to both the level and the ranking positions. This impression is confirmed by corresponding correlation coefficients (see Spearman Rank and Pearson coefficients in Table 2), which are all below 0.7. The results indicate that interpretations may differ slightly depending on the year selected.

**Table 2:** Spearman rank and Pearson correlations across time for the graduation shift.

	Spearman Rank Correlation				Pearson Correlation		
	**2008**	**2009**	**2010**		**2008**	**2009**	**2010**
**2008**	1.000			**2008**	1.000		
**2009**	0.658	1.000		**2009**	0.698	1.000	
**2010**	0.476	0.674	1.000	**2010**	0.530	0.670	1.000

**Fig 3:**
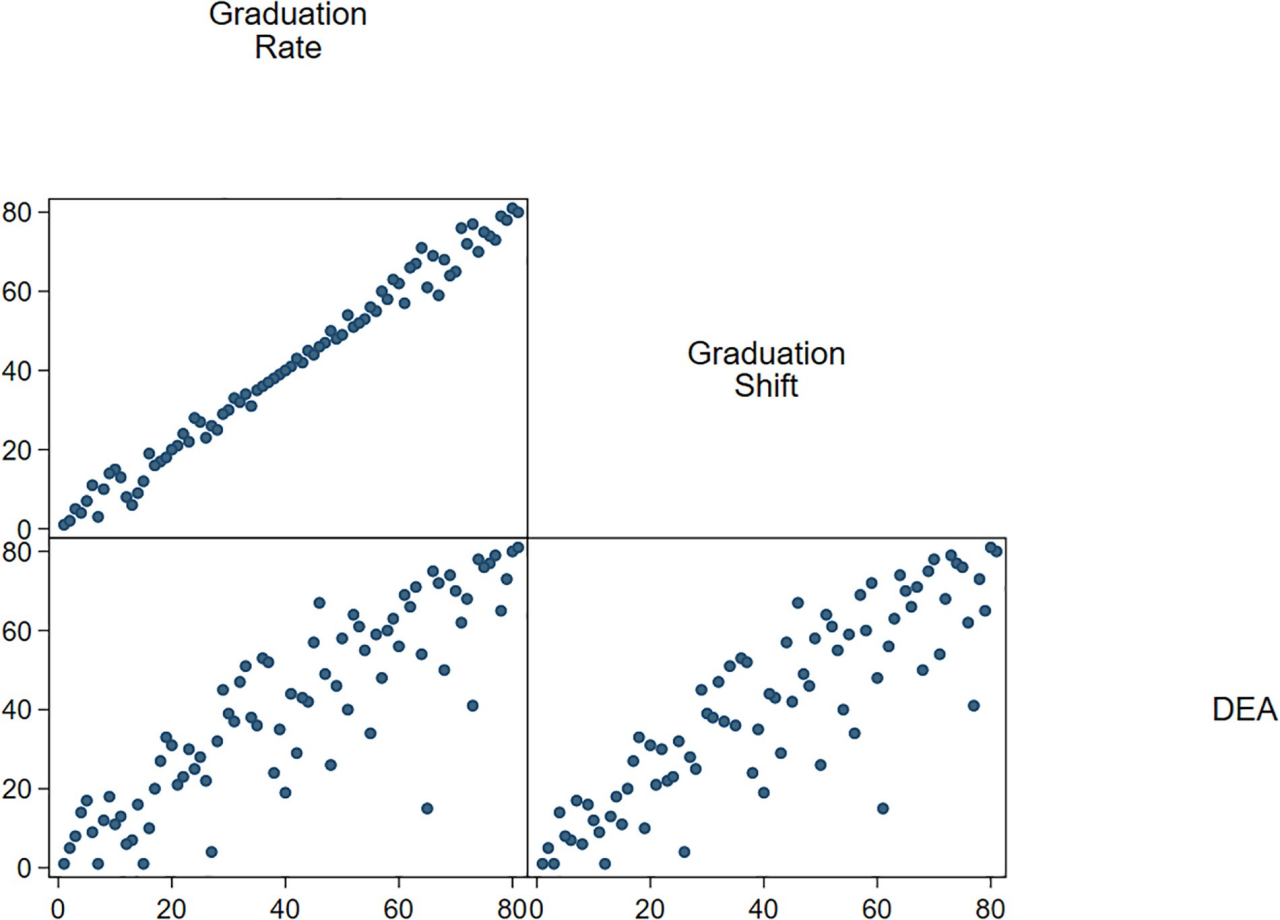
Ranking comparison of different approaches for measuring efficiency (2010).

Fig 3 shows the scatterplot of the ranking positions, which result from the different approaches for 2010. The scatterplot reveals that the ranking positions of the universities are fairly homogenous when we compare the graduation rate with the graduation shift. This is confirmed by the results in Table 3, which provide the Spearman rank and Pearson correlations for the different comparisons across all years. The coefficients for the correlation between graduation rate and shift always exceed 0.96. Thus, both approaches lead to quite similar conclusions for most of the universities.

**Table 3:** Correlations between different efficiency measures.

	Spearman Rank Correlations			Pearson Correlation		
2008	Graduation Rate	Graduation Shift	DEA	Graduation Rate	Graduation Shift	DEA
**Graduation Rate**	1.000			1.000		
**Graduation Shift**	0.989	1.000		0.966	1.000	
**DEA**	0.877	0.861	1.000	0.883	0.872	1.000
2009						
**Graduation Rate**	1.000			1.000		
**Graduation Shift**	0.991	1.000		0.978	1.000	
**DEA**	0.822	0.796	1.000	0.871	0.844	1.000
2010						
**Graduation Rate**	1.000			1.000		
**Graduation Shift**	0.993	1.000		0.979	1.000	
**DEA**	0.880	0.872	1.000	0.881	0.866	1.000

Tables 4 to 6 document the results of the analyses for the individual universities of applied sciences for the years 2008–2010. The tables are sorted in alphabetical order. The tables show the expenditure and the number of first semester students and graduates–including their relative shares. The graduation shift is the difference between the two relative efficiency measures %*S*/%*E* and %*G*/%*E*. In the last column, DEA scores are listed. With an average of 0.739 for the year 2010 (see Table 6) the institutions exhibit a fairly high efficiency level. The University of Applied Sciences in Neu-Ulm has the highest graduation shift compared to the other universities. In other words, the university exhibits the best relative graduation process of students based on the given expenses. While this university is only ranked 60^th^ with respect to the relative student efficiency (%*S*/%*E*), it reaches the seventh position when it comes to graduation efficiency (%*G*/%*E*). This results in a very good relative performance with respect to the graduation shift. A DEA score of 1.00 and the first rank with respect to the graduation rate confirm the high graduation efficiency. At the lower end of the ranking we find the FH Brandenburg, which performs quite well with respect to student efficiency (rank 10), but drops to the 63^rd^ position in the graduation ranking. In addition, the university features a very small DEA score and graduation rate.

**Table 4:** Input and output indicators for 81 universities of applied sciences and the resulting graduation shift (and DEA) for the year 2008.

	E	%E	S	G	%S	G/S	%S/%E	(*G*/*S*)/%*E*	Graduation Shift	DEA
FH Aachen	60,563	0.02	1,887	1,555	0.02	0.02	0.76	0.79	0.03	0.760
FH Aalen	24,513	0.01	840	714	0.01	0.01	0.84	0.90	0.06	0.722
FH Albstadt-Sigmaringen	14,874	0.01	527	582	0.01	0.01	0.86	1.20	0.34	0.920
FH Amberg-Weiden	11,846	0.00	807	482	0.01	0.01	1.66	1.25	-0.41	0.618
FH Anhalt	48,808	0.02	1,795	1,273	0.02	0.02	0.90	0.80	-0.09	0.662
FH Ansbach	10,290	0.00	494	355	0.00	0.00	1.17	1.06	-0.11	0.728
FH Augsburg	22,072	0.01	1,122	1,037	0.01	0.01	1.24	1.45	0.21	0.887
FH Bielefeld	37,422	0.01	1,482	1,137	0.01	0.01	0.97	0.93	-0.03	0.705
FH Bingen	12,466	0.00	639	430	0.01	0.01	1.25	1.06	-0.19	0.637
FH Bochum	31,536	0.01	901	873	0.01	0.01	0.70	0.85	0.16	0.825
FH Bonn-Rhein-Sieg	26,429	0.01	1,228	935	0.01	0.01	1.13	1.09	-0.04	0.719
FH Brandenburg	13,350	0.01	829	411	0.01	0.01	1.51	0.95	-0.57	0.491
FH Braunschweig-Wolfenbüttel	48,239	0.02	1,857	1,496	0.02	0.02	0.94	0.95	0.02	0.758
FH Coburg	18,355	0.01	909	744	0.01	0.01	1.21	1.25	0.04	0.753
FH Darmstadt	59,731	0.02	2,668	1,893	0.03	0.02	1.09	0.98	-0.11	0.732
FH Deggendorf	15,860	0.01	998	834	0.01	0.01	1.53	1.62	0.08	0.835
FH Dortmund	46,333	0.02	1,647	1,300	0.02	0.02	0.87	0.86	0.00	0.726
FH Düsseldorf	43,591	0.02	1,687	1,319	0.02	0.02	0.94	0.93	-0.01	0.727
FH Eberswalde	13,825	0.01	574	380	0.01	0.00	1.01	0.85	-0.17	0.580
FH Erfurt	29,152	0.01	1,395	1,034	0.01	0.01	1.17	1.09	-0.08	0.719
FH Flensburg	16,470	0.01	838	544	0.01	0.01	1.24	1.02	-0.22	0.603
FH Frankfurt a.M.	47,840	0.02	1,822	1,640	0.02	0.02	0.93	1.05	0.13	0.844
FH Fulda	29,075	0.01	1,289	916	0.01	0.01	1.08	0.97	-0.11	0.665
FH Furtwangen	28,173	0.01	817	917	0.01	0.01	0.71	1.00	0.29	0.952
FH Gelsenkirchen	47,248	0.02	1,732	1,036	0.02	0.01	0.89	0.67	-0.22	0.555
FH Hannover	49,316	0.02	1,441	1,326	0.01	0.02	0.71	0.83	0.11	0.824
FH Harz	14,707	0.01	791	489	0.01	0.01	1.31	1.02	-0.29	0.586
FH Heilbronn	30,607	0.01	1,113	962	0.01	0.01	0.89	0.97	0.08	0.749
FH Hildesheim-Holzminden-Göttingen	38,664	0.02	1,327	1,189	0.01	0.01	0.84	0.95	0.11	0.796
FH Hof	11,560	0.00	611	521	0.01	0.01	1.29	1.39	0.10	0.838
FH Ingolstadt	15,532	0.01	715	646	0.01	0.01	1.12	1.28	0.16	0.815
FH Jena	27,265	0.01	1,202	887	0.01	0.01	1.07	1.00	-0.07	0.683
FH Kaiserslautern	34,948	0.01	1,374	948	0.01	0.01	0.96	0.83	-0.12	0.627
FH Karlsruhe	36,101	0.01	1,322	1,276	0.01	0.02	0.89	1.09	0.19	0.862
FH Kempten	14,614	0.01	892	677	0.01	0.01	1.49	1.43	-0.06	0.745
FH Kiel	24,818	0.01	1,202	1,027	0.01	0.01	1.18	1.27	0.09	0.816
FH Koblenz	36,663	0.01	1,466	1,291	0.01	0.02	0.97	1.08	0.11	0.810
FH Konstanz	24,923	0.01	801	831	0.01	0.01	0.78	1.03	0.24	0.879
FH Köln	107,938	0.04	3,385	2,646	0.03	0.03	0.76	0.75	-0.01	0.807
FH Landshut	12,526	0.00	974	931	0.01	0.01	1.90	2.29	0.39	1.000
FH Lübeck	22,043	0.01	965	632	0.01	0.01	1.07	0.88	-0.19	0.585
FH Magdeburg-Stendal	31,520	0.01	1,457	1,037	0.01	0.01	1.13	1.01	-0.11	0.686
FH Merseburg	21,687	0.01	839	681	0.01	0.01	0.94	0.97	0.02	0.697
FH München	72,024	0.03	3,458	2,287	0.03	0.03	1.17	0.98	-0.19	0.791
FH Münster	63,243	0.03	1,955	2,114	0.02	0.03	0.75	1.03	0.27	1.000
FH Neu-Ulm	8,446	0.00	486	415	0.00	0.01	1.40	1.51	0.11	1.000
FH Neubrandenburg	17,856	0.01	515	588	0.00	0.01	0.70	1.01	0.31	0.947
FH Niederrhein	55,327	0.02	2,406	1,721	0.02	0.02	1.06	0.96	-0.10	0.707
FH Nordhausen	10,449	0.00	658	405	0.01	0.00	1.54	1.19	-0.34	0.656
FH Nürnberg	41,496	0.02	2,163	1,786	0.02	0.02	1.27	1.32	0.05	0.917
FH Nürtingen	17,837	0.01	799	809	0.01	0.01	1.09	1.40	0.30	0.900
FH Osnabrück	55,247	0.02	2,437	1,877	0.02	0.02	1.08	1.05	-0.03	0.772
FH Ostwestfalen-Lippe	44,464	0.02	1,212	861	0.01	0.01	0.66	0.60	-0.07	0.622
FH Pforzheim	26,738	0.01	878	950	0.01	0.01	0.80	1.09	0.29	0.920
FH Potsdam	17,757	0.01	675	499	0.01	0.01	0.93	0.86	-0.06	0.629
FH Ravensburg-Weingarten	14,373	0.01	537	566	0.01	0.01	0.91	1.21	0.30	0.888
FH Regensburg	30,540	0.01	1,461	1,374	0.01	0.02	1.17	1.38	0.22	0.917
FH RheinMain	55,234	0.02	1,924	1,585	0.02	0.02	0.85	0.88	0.03	0.771
FH Rosenheim	20,918	0.01	1,059	763	0.01	0.01	1.23	1.12	-0.11	0.684
FH Schmalkalden	13,942	0.01	802	727	0.01	0.01	1.40	1.60	0.20	0.878
FH Stralsund	17,076	0.01	657	498	0.01	0.01	0.94	0.90	-0.04	0.645
FH Trier	43,647	0.02	1,594	907	0.02	0.01	0.89	0.64	-0.25	0.522
FH Ulm	21,389	0.01	818	720	0.01	0.01	0.93	1.04	0.10	0.754
FH Weihenstephan-Triesdorf	27,114	0.01	1,097	776	0.01	0.01	0.99	0.88	-0.11	0.630
FH Westküste, Heide	7,671	0.00	389	265	0.00	0.00	1.24	1.06	-0.17	0.736
FH Wismar	29,551	0.01	1,605	911	0.02	0.01	1.32	0.95	-0.38	0.596
FH Würzburg-Schweinfurt	30,904	0.01	1,867	1,268	0.02	0.02	1.47	1.26	-0.21	0.805
FH Zittau/Görlitz	29,711	0.01	949	697	0.01	0.01	0.78	0.72	-0.06	0.626
FH für Technik Stuttgart	19,227	0.01	672	644	0.01	0.01	0.85	1.03	0.18	0.806
FH für Technik und Wirtschaft Berlin	54,394	0.02	2,049	2,121	0.02	0.03	0.92	1.20	0.28	0.983
FH für Technik und Wirtschaft Dresden	38,181	0.02	1,480	1,108	0.01	0.01	0.95	0.89	-0.05	0.684
FH für Technik und Wirtschaft Offenburg	17,573	0.01	696	506	0.01	0.01	0.97	0.89	-0.08	0.623
FH für Technik und Wirtschaft, Reutlingen	28,562	0.01	1,038	1,214	0.01	0.01	0.89	1.31	0.42	1.000
FH für Technik, Wirtschaft und Kultur Leipzig	34,960	0.01	1,719	1,275	0.02	0.02	1.20	1.12	-0.08	0.743
H Bremen	36,781	0.01	2,061	1,353	0.02	0.02	1.37	1.13	-0.23	0.759
H Bremerhaven	15,228	0.01	730	499	0.01	0.01	1.17	1.01	-0.16	0.625
H f. Technik u. Wirtsch. d. Saarlandes Saarbrücken	24,678	0.01	1,402	673	0.01	0.01	1.39	0.84	-0.55	0.496
Technische FH Berlin	63,879	0.03	1,981	1,966	0.02	0.02	0.76	0.95	0.19	0.921
Technische FH Wildau	18,709	0.01	1,213	917	0.01	0.01	1.58	1.51	-0.07	0.799
Technische Hochschule Mittelhessen, FH (THM)	52,762	0.02	2,263	1,732	0.02	0.02	1.05	1.01	-0.04	0.750
Westsächsische H Zwickau	36,445	0.01	1,309	932	0.01	0.01	0.88	0.79	-0.09	0.634
	2,527,814		103,675	82,143						0.754

**Table 5:** Input and output indicators for 81 universities of applied sciences and the resulting graduation shift (and DEA) for the year 2009.

	E	%E	S	G	%S	G/S	%S/%E	(*G*/*S*)/%*E*	Graduation Shift	DEA
FH Aachen	67,074	0.02	2,117	1,596	0.02	0.02	0.76	0.76	0.00	0.710
FH Aalen	27,563	0.01	1,081	1,164	0.01	0.01	0.95	1.35	0.40	0.948
FH Albstadt-Sigmaringen	16,083	0.01	598	623	0.01	0.01	0.90	1.24	0.34	0.879
FH Amberg-Weiden	13,720	0.00	823	450	0.01	0.01	1.45	1.05	-0.40	0.536
FH Anhalt	50,761	0.02	1,541	1,354	0.01	0.02	0.74	0.85	0.12	0.793
FH Ansbach	12,079	0.00	562	404	0.00	0.00	1.13	1.07	-0.06	0.670
FH Augsburg	23,768	0.01	1,207	994	0.01	0.01	1.23	1.34	0.11	0.799
FH Bielefeld	42,530	0.02	1,631	1,248	0.01	0.01	0.93	0.94	0.01	0.708
FH Bingen	14,192	0.01	673	428	0.01	0.00	1.15	0.96	-0.18	0.581
FH Bochum	34,270	0.01	1,077	815	0.01	0.01	0.76	0.76	0.00	0.651
FH Bonn-Rhein-Sieg	29,975	0.01	1,308	1,064	0.01	0.01	1.06	1.14	0.08	0.759
FH Brandenburg	14,841	0.01	929	437	0.01	0.01	1.52	0.94	-0.57	0.465
FH Braunschweig-Wolfenbüttel	53,648	0.02	2,099	1,470	0.02	0.02	0.95	0.88	-0.07	0.669
FH Coburg	20,851	0.01	1,026	748	0.01	0.01	1.19	1.15	-0.04	0.683
FH Darmstadt	63,603	0.02	2,983	2,015	0.03	0.02	1.14	1.01	-0.12	0.742
FH Deggendorf	19,019	0.01	1,169	828	0.01	0.01	1.49	1.39	-0.10	0.722
FH Dortmund	52,027	0.02	1,739	1,326	0.02	0.02	0.81	0.82	0.01	0.702
FH Düsseldorf	47,255	0.02	1,872	1,192	0.02	0.01	0.96	0.81	-0.15	0.601
FH Eberswalde	15,002	0.01	637	384	0.01	0.00	1.03	0.82	-0.21	0.530
FH Erfurt	28,036	0.01	1,455	1,019	0.01	0.01	1.26	1.16	-0.09	0.701
FH Flensburg	17,701	0.01	885	751	0.01	0.01	1.21	1.36	0.15	0.782
FH Frankfurt a.M.	51,786	0.02	2,116	1,580	0.02	0.02	0.99	0.98	-0.01	0.717
FH Fulda	31,139	0.01	1,470	1,028	0.01	0.01	1.14	1.06	-0.09	0.680
FH Furtwangen	31,687	0.01	1,166	909	0.01	0.01	0.89	0.92	0.03	0.682
FH Gelsenkirchen	50,978	0.02	1,999	929	0.02	0.01	0.95	0.58	-0.37	0.442
FH Hannover	52,956	0.02	1,605	1,437	0.01	0.02	0.73	0.87	0.13	0.812
FH Harz	16,985	0.01	892	648	0.01	0.01	1.27	1.22	-0.05	0.680
FH Heilbronn	36,316	0.01	1,364	1,076	0.01	0.01	0.91	0.95	0.04	0.710
FH Hildesheim-Holzminden-Göttingen	40,928	0.01	1,313	1,258	0.01	0.01	0.78	0.98	0.21	0.848
FH Hof	13,337	0.00	747	455	0.01	0.01	1.36	1.09	-0.26	0.587
FH Ingolstadt	16,949	0.01	908	650	0.01	0.01	1.30	1.23	-0.07	0.674
FH Jena	29,049	0.01	1,377	962	0.01	0.01	1.15	1.06	-0.09	0.675
FH Kaiserslautern	38,342	0.01	1,366	963	0.01	0.01	0.86	0.80	-0.06	0.631
FH Karlsruhe	40,787	0.01	1,527	1,194	0.01	0.01	0.91	0.94	0.03	0.715
FH Kempten	18,309	0.01	995	636	0.01	0.01	1.32	1.11	-0.20	0.614
FH Kiel	26,896	0.01	1,343	1,319	0.01	0.02	1.21	1.57	0.36	0.961
FH Koblenz	41,162	0.01	1,444	1,358	0.01	0.02	0.85	1.06	0.21	0.848
FH Konstanz	27,694	0.01	915	895	0.01	0.01	0.80	1.03	0.23	0.833
FH Köln	119,071	0.04	3,484	2,719	0.03	0.03	0.71	0.73	0.02	0.830
FH Landshut	15,168	0.01	988	702	0.01	0.01	1.58	1.48	-0.10	0.715
FH Lübeck	22,824	0.01	984	817	0.01	0.01	1.04	1.15	0.10	0.741
FH Magdeburg-Stendal	33,649	0.01	1,548	1,109	0.01	0.01	1.11	1.05	-0.06	0.694
FH Merseburg	23,026	0.01	845	601	0.01	0.01	0.89	0.84	-0.05	0.606
FH München	80,837	0.03	3,622	2,527	0.03	0.03	1.09	1.00	-0.08	0.821
FH Münster	70,061	0.03	2,166	2,026	0.02	0.02	0.75	0.93	0.18	0.885
FH Neu-Ulm	10,395	0.00	537	505	0.00	0.01	1.25	1.55	0.30	0.964
FH Neubrandenburg	18,779	0.01	609	524	0.01	0.01	0.79	0.89	0.11	0.720
FH Niederrhein	62,065	0.02	2,313	1,831	0.02	0.02	0.90	0.94	0.04	0.761
FH Nordhausen	11,038	0.00	834	489	0.01	0.01	1.83	1.42	-0.41	0.619
FH Nürnberg	45,807	0.02	2,350	1,777	0.02	0.02	1.24	1.24	0.00	0.847
FH Nürtingen	20,240	0.01	870	997	0.01	0.01	1.04	1.58	0.54	1.000
FH Osnabrück	64,019	0.02	2,671	2,422	0.02	0.03	1.01	1.21	0.20	0.894
FH Ostwestfalen-Lippe	48,343	0.02	1,638	967	0.01	0.01	0.82	0.64	-0.18	0.540
FH Pforzheim	30,254	0.01	1,006	1,034	0.01	0.01	0.81	1.09	0.29	0.878
FH Potsdam	18,639	0.01	712	583	0.01	0.01	0.93	1.00	0.08	0.698
FH Ravensburg-Weingarten	16,058	0.01	604	540	0.01	0.01	0.91	1.08	0.17	0.756
FH Regensburg	35,599	0.01	1,903	1,334	0.02	0.02	1.29	1.20	-0.10	0.766
FH RheinMain	60,717	0.02	2,079	1,715	0.02	0.02	0.83	0.90	0.07	0.777
FH Rosenheim	23,537	0.01	1,204	785	0.01	0.01	1.24	1.07	-0.17	0.633
FH Schmalkalden	14,591	0.01	828	692	0.01	0.01	1.37	1.52	0.14	0.805
FH Stralsund	18,079	0.01	771	494	0.01	0.01	1.03	0.87	-0.16	0.560
FH Trier	48,549	0.02	1,797	981	0.02	0.01	0.90	0.65	-0.25	0.510
FH Ulm	23,427	0.01	874	804	0.01	0.01	0.90	1.10	0.19	0.786
FH Weihenstephan-Triesdorf	30,369	0.01	1,292	1,083	0.01	0.01	1.03	1.14	0.11	0.774
FH Westküste, Heide	8,507	0.00	365	238	0.00	0.00	1.04	0.90	-0.14	0.580
FH Wismar	32,025	0.01	2,082	1,159	0.02	0.01	1.57	1.16	-0.42	0.718
FH Würzburg-Schweinfurt	35,431	0.01	1,999	1,343	0.02	0.02	1.37	1.21	-0.15	0.774
FH Zittau/Görlitz	31,056	0.01	953	785	0.01	0.01	0.74	0.81	0.07	0.702
FH für Technik Stuttgart	21,875	0.01	805	713	0.01	0.01	0.89	1.04	0.15	0.754
FH für Technik und Wirtschaft Berlin	58,638	0.02	2,429	2,359	0.02	0.03	1.00	1.29	0.28	0.947
FH für Technik und Wirtschaft Dresden	40,145	0.01	1,468	986	0.01	0.01	0.89	0.79	-0.10	0.610
FH für Technik und Wirtschaft Offenburg	20,952	0.01	945	637	0.01	0.01	1.09	0.97	-0.12	0.606
FH für Technik und Wirtschaft, Reutlingen	31,914	0.01	1,201	1,133	0.01	0.01	0.91	1.14	0.22	0.832
FH für Technik, Wirtschaft und Kultur Leipzig	37,375	0.01	1,919	1,426	0.02	0.02	1.24	1.22	-0.02	0.791
H Bremen	38,622	0.01	2,205	1,543	0.02	0.02	1.38	1.28	-0.10	0.836
H Bremerhaven	15,889	0.01	889	450	0.01	0.01	1.35	0.91	-0.45	0.484
H f. Technik u. Wirtsch. d. Saarlandes Saarbrücken	28,436	0.01	1,471	861	0.01	0.01	1.25	0.97	-0.28	0.586
Technische FH Berlin	67,159	0.02	2,267	1,878	0.02	0.02	0.82	0.89	0.08	0.791
Technische FH Wildau	19,955	0.01	1,425	939	0.01	0.01	1.73	1.51	-0.22	0.788
Technische Hochschule Mittelhessen, FH (THM)	58,891	0.02	2,494	1,827	0.02	0.02	1.03	0.99	-0.03	0.719
Westsächsische H Zwickau	38,203	0.01	1,376	931	0.01	0.01	0.87	0.78	-0.09	0.607
	2,779,505		114,781	86,873						0.722

**Table 6:** Input and output indicators for 81 universities of applied sciences and the resulting graduation shift (and DEA) for the year 2010.

	E	%E	S	G	%S	G/S	%S/%E	(*G*/*S*)/%*E*	Graduation Shift	DEA
FH Aachen	73,983	0.02	2,133	1,572	0.02	0.74	0.74	30.09	-0.04	0.695
FH Aalen	30,380	0.01	1,201	881	0.01	0.73	1.02	72.95	-0.06	0.657
FH Albstadt-Sigmaringen	17,438	0.01	584	602	0.00	1.03	0.86	178.58	0.27	0.861
FH Amberg-Weiden	16,323	0.01	775	526	0.01	0.68	1.22	125.61	-0.16	0.617
FH Anhalt	55,005	0.02	1,744	1,412	0.01	0.81	0.81	44.47	0.03	0.741
FH Ansbach	14,006	0.00	596	485	0.01	0.81	1.09	175.53	0.04	0.719
FH Augsburg	25,812	0.01	1,209	1,177	0.01	0.97	1.20	113.94	0.29	0.920
FH Bielefeld	47,318	0.02	1,820	1,366	0.02	0.75	0.99	47.92	-0.04	0.704
FH Bingen	15,167	0.01	667	442	0.01	0.66	1.13	131.99	-0.17	0.589
FH Bochum	37,153	0.01	1,363	785	0.01	0.58	0.94	46.83	-0.25	0.517
FH Bonn-Rhein-Sieg	33,806	0.01	1,362	1,097	0.01	0.81	1.03	71.98	0.03	0.737
FH Brandenburg	16,120	0.01	851	440	0.01	0.52	1.36	96.90	-0.46	0.486
FH Braunschweig-Wolfenbüttel	57,484	0.02	2,329	2,293	0.02	0.98	1.04	51.74	0.27	0.954
FH Coburg	24,231	0.01	1,090	778	0.01	0.71	1.16	88.99	-0.10	0.656
FH Darmstadt	67,821	0.02	2,875	1,901	0.02	0.66	1.09	29.45	-0.17	0.678
FH Deggendorf	22,282	0.01	1,181	860	0.01	0.73	1.36	98.73	-0.09	0.713
FH Dortmund	56,610	0.02	1,804	1,338	0.02	0.74	0.82	39.58	-0.04	0.682
FH Düsseldorf	51,427	0.02	1,800	1,363	0.02	0.76	0.90	44.48	-0.03	0.703
FH Eberswalde	16,271	0.01	575	429	0.00	0.75	0.91	138.53	-0.04	0.624
FH Erfurt	28,526	0.01	1,657	939	0.01	0.57	1.49	60.01	-0.41	0.629
FH Flensburg	18,760	0.01	788	573	0.01	0.73	1.08	117.10	-0.08	0.631
FH Frankfurt a.M.	55,281	0.02	2,174	1,492	0.02	0.69	1.01	37.50	-0.12	0.659
FH Fulda	33,526	0.01	1,599	1,011	0.01	0.63	1.23	56.97	-0.23	0.622
FH Furtwangen	35,307	0.01	1,141	1,068	0.01	0.94	0.83	80.09	0.16	0.812
FH Gelsenkirchen	54,716	0.02	2,070	929	0.02	0.45	0.97	24.78	-0.41	0.427
FH Hannover	57,281	0.02	1,596	1,583	0.01	0.99	0.72	52.31	0.19	0.899
FH Harz	19,033	0.01	673	619	0.01	0.92	0.91	145.99	0.16	0.774
FH Heilbronn	41,420	0.01	1,419	1,331	0.01	0.94	0.88	68.41	0.18	0.841
FH Hildesheim-Holzminden-Göttingen	42,706	0.01	1,293	1,328	0.01	1.03	0.78	72.65	0.24	0.907
FH Hof	15,422	0.01	738	535	0.01	0.72	1.23	142.01	-0.09	0.662
FH Ingolstadt	20,291	0.01	973	663	0.01	0.68	1.23	101.45	-0.16	0.628
FH Jena	30,334	0.01	1,274	882	0.01	0.69	1.08	68.95	-0.12	0.636
FH Kaiserslautern	40,533	0.01	1,430	981	0.01	0.69	0.91	51.13	-0.11	0.618
FH Karlsruhe	44,929	0.01	1,767	1,416	0.02	0.80	1.01	53.88	0.03	0.751
FH Kempten	21,634	0.01	1,204	773	0.01	0.64	1.43	89.65	-0.26	0.640
FH Kiel	29,343	0.01	1,356	1,192	0.01	0.88	1.19	90.50	0.15	0.840
FH Koblenz	43,412	0.01	1,404	1,385	0.01	0.99	0.83	68.65	0.22	0.880
FH Konstanz	29,957	0.01	942	1,012	0.01	1.07	0.81	108.34	0.30	0.916
FH Köln	129,104	0.04	4,246	2,840	0.04	0.67	0.84	15.65	-0.12	0.867
FH Landshut	17,859	0.01	1,044	700	0.01	0.67	1.50	113.42	-0.21	0.662
FH Lübeck	24,269	0.01	1,091	834	0.01	0.76	1.15	95.16	-0.03	0.703
FH Magdeburg-Stendal	35,268	0.01	1,490	1,271	0.01	0.85	1.09	73.07	0.10	0.799
FH Merseburg	23,797	0.01	801	494	0.01	0.62	0.86	78.29	-0.18	0.523
FH München	90,391	0.03	3,385	3,277	0.03	0.97	0.96	32.36	0.23	1.000
FH Münster	77,227	0.03	2,240	2,114	0.02	0.94	0.75	36.92	0.15	0.899
FH Neu-Ulm	12,702	0.00	434	529	0.00	1.22	0.88	289.91	0.49	1.000
FH Neubrandenburg	19,595	0.01	652	526	0.01	0.81	0.85	124.38	0.03	0.678
FH Niederrhein	69,262	0.02	2,444	1,900	0.02	0.78	0.91	33.91	-0.01	0.754
FH Nordhausen	11,378	0.00	631	526	0.01	0.83	1.42	221.33	0.09	0.835
FH Nürnberg	53,348	0.02	2,777	2,306	0.02	0.83	1.34	47.02	0.08	0.976
FH Nürtingen	22,445	0.01	979	948	0.01	0.97	1.12	130.34	0.27	0.866
FH Osnabrück	70,355	0.02	3,018	2,355	0.03	0.78	1.10	33.51	0.00	0.825
FH Ostwestfalen-Lippe	51,037	0.02	1,499	993	0.01	0.66	0.75	39.21	-0.12	0.596
FH Pforzheim	33,067	0.01	1,113	1,023	0.01	0.92	0.86	83.97	0.15	0.794
FH Potsdam	19,360	0.01	713	586	0.01	0.82	0.95	128.25	0.05	0.697
FH Ravensburg-Weingarten	17,567	0.01	622	649	0.01	1.04	0.91	179.44	0.30	0.875
FH Regensburg	40,689	0.01	2,012	1,537	0.02	0.76	1.27	56.72	-0.03	0.801
FH RheinMain	64,206	0.02	2,253	1,459	0.02	0.65	0.90	30.47	-0.15	0.618
FH Rosenheim	26,460	0.01	1,288	913	0.01	0.71	1.25	80.93	-0.12	0.684
FH Schmalkalden	15,159	0.01	816	677	0.01	0.83	1.38	165.35	0.08	0.786
FH Stralsund	18,818	0.01	705	552	0.01	0.78	0.96	125.70	0.00	0.665
FH Trier	50,807	0.02	1,629	1,065	0.01	0.65	0.82	38.87	-0.13	0.594
FH Ulm	24,630	0.01	877	708	0.01	0.81	0.91	99.02	0.03	0.686
FH Weihenstephan-Triesdorf	33,255	0.01	1,388	972	0.01	0.70	1.07	63.62	-0.11	0.649
FH Westküste, Heide	9,230	0.00	336	263	0.00	0.78	0.94	256.19	0.00	0.673
FH Wismar	34,043	0.01	1,810	1,302	0.02	0.72	1.37	63.83	-0.11	0.772
FH Würzburg-Schweinfurt	40,040	0.01	2,096	1,548	0.02	0.74	1.34	55.72	-0.07	0.816
FH Zittau/Görlitz	32,839	0.01	890	896	0.01	1.01	0.70	92.61	0.20	0.856
FH für Technik Stuttgart	24,179	0.01	722	829	0.01	1.15	0.77	143.46	0.36	0.969
FH für Technik und Wirtschaft Berlin	62,939	0.02	2,660	2,700	0.02	1.02	1.09	48.72	0.32	1.000
FH für Technik und Wirtschaft Dresden	41,380	0.01	1,564	906	0.01	0.58	0.97	42.29	-0.25	0.532
FH für Technik und Wirtschaft Offenburg	24,565	0.01	1,013	735	0.01	0.73	1.06	89.23	-0.08	0.642
FH für Technik und Wirtschaft, Reutlingen	35,004	0.01	1,151	1,167	0.01	1.01	0.84	87.50	0.25	0.881
FH für Technik, Wirtschaft und Kultur Leipzig	39,531	0.01	1,751	1,466	0.01	0.84	1.14	63.98	0.08	0.811
H Bremen	40,240	0.01	2,107	1,617	0.02	0.77	1.35	57.62	-0.02	0.849
H Bremerhaven	16,462	0.01	763	470	0.01	0.62	1.19	113.04	-0.25	0.555
H f. Technik u. Wirtsch. d. Saarlandes Saarbrücken	31,193	0.01	1,675	942	0.01	0.56	1.38	54.47	-0.39	0.594
Technische FH Berlin	70,384	0.02	2,338	2,023	0.02	0.87	0.85	37.14	0.09	0.832
Technische FH Wildau	21,383	0.01	1,426	880	0.01	0.62	1.71	87.19	-0.36	0.709
Technische Hochschule Mittelhessen, FH (THM)	64,858	0.02	2,443	2,058	0.02	0.84	0.97	39.24	0.07	0.817
Westsächsische H Zwickau	39,660	0.01	1,259	940	0.01	0.75	0.82	56.87	-0.04	0.657
	3,021,027		117,608	91,955						0.739

**Fig 4:**
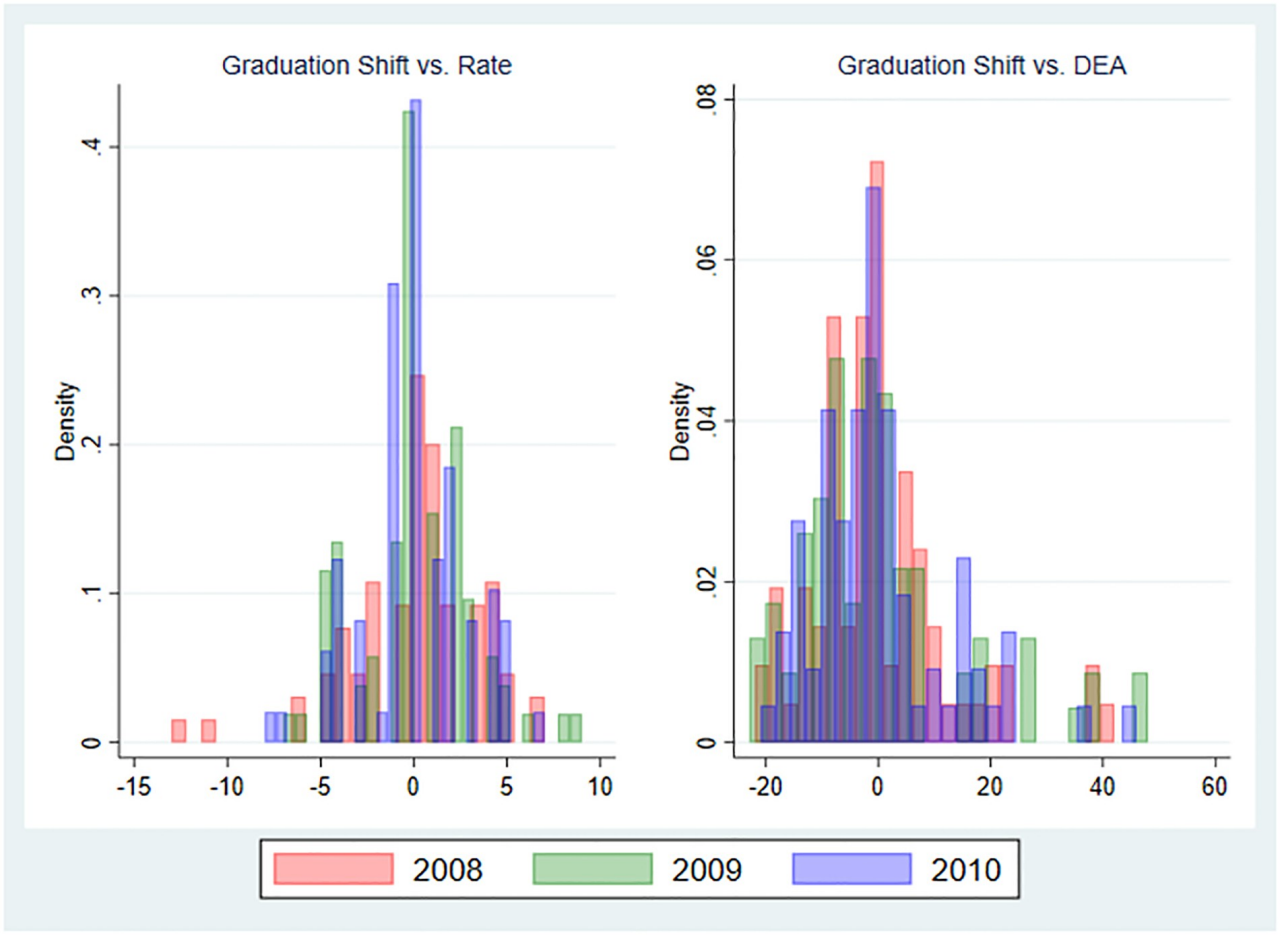
Histogram of ranking differences between efficiency approaches across years.

To demonstrate differences between graduation rate and shift, we plot a histogram depicting the ranking differences for all three years (see Fig 4). The histograms show the difference in ranking positions between the graduation shift and the graduation rate and DEA, respectively, for each year. The left Panel of Fig 4 reports the differences between the graduation shift and rate. It shows that there are more universities where the ranking position differs than the high Spearman correlation coefficient might suggest. The average ranking change over all three years is about 2.25 ranking positions. The maximum difference between the graduation rate and shift rankings is 13 positions in 2008: the University of Applied Science in Landshut is ranked 2^nd^ with respect to the graduation shift but drops to the 15^th^ place in the graduation rate ranking. Evaluating the positions of the universities in more detail, it can be noted that the first 2 positions in both rankings remain the same over all three years. However, there is some variation for universities which are listed in the top 10 of the respective ranking. Some universities enter the top 10 when we account the expenditures. This sensitivity of the ranking should not be underrated, since rankings have implications for the reputation of a university.

We have two possible explanations for our result that graduation shift and rate lead to similar rankings, but exhibit some differences for selected universities especially at the upper and lower part of the ranking positions (see Fig 3): firstly, graduation rates are good proxies for teaching efficiency, even if they are adjusted to relative expenditure figures. Secondly, we have a quite homogeneous sample including similar universities of applied sciences.

The correlations between the results of the graduation shift and DEA are fairly high with around 0.85, but more dispersed. However, since the correlation is not perfect, a high DEA score is not necessarily associated with a high graduation shift. This is supported by the ranking differences shown in the right panel of Fig 4. The differences are larger than those between the graduation shift and rate are. The average positional change across the years is about nine. The largest gain is 48 positions and the largest drop is 23. The Technical University of Applied Sciences in Cologne, for example, is ranked 15^th^ in the DEA ranking in 2010, but only 61^st^ in the graduation shift ranking. Hence, the figure shows that the assessment of teaching efficiency differs between the standard DEA approach and the newly introduced graduation shift.

## Sensitivity analysis

In order to test the robustness of our results we use the number of employees as an alternative input measure (instead of expenditures). The number of employees is a frequently used input indicator at the institutional level [2]. We refrain from reporting results on the comparison of this graduation shift variant to the graduation rate and DEA. We concentrate on the comparison between the two graduation shift variants (using number of employees and expenditures as inputs). Table 7 shows the correlations between the two variants. The correlations of the ranks (according to the spearman rank correlation) and the values (according to the Pearson correlation) are nearly perfect, always exceeding r = 0.9 across the years. The comparison shows therefore that the conclusions on the universities remain nearly the same independent of the used input measure. However, since the personnel expenditures account for the majority of the overall institutional costs, this result is not surprising.

**Table 7:** Spearman rank and Pearson correlations for the expenditures and employee variation.

	Spearman Rank Correlation	Pearson Correlation
**2008**	0.994	0.990
**2009**	0.991	0.991
**2010**	0.995	0.964

## Limitations

No indicator is without limitations. We stated above that the graduation shift can handle only one input and two outputs. The DEA might be the better alternative, if this is not sufficient in the statistical analysis.

Although the graduation shift yields plausible results in our study and correlates highly with other efficiency measures, the shift has some drawbacks, which are illustrated in Table 8. The table features some examples with three artificial universities and their corresponding inputs and outputs. Panel A is the starting point where all indicators are identical. The graduation shifts of the universities are zero. In Panel B we increase ceteris paribus the expenditure of university A which leaves the graduation shift unchanged. One would expect a decrease. In Panel C–with identical expenditures and numbers of students as in Panel A–we drop the graduation rate of university C, which results in a negative graduation shift for C and positive graduation shifts for A and B. In Panel D, we additionally lower the expenditures of university B, which leads to the highest graduation shift score among the universities. The results in Panels C and D are unsurprising. In Panels E and F, however, we see the opposite effect, which defies our expectations. In Panel E, we have two universities (B and C) with a negative graduation shift due to smaller graduation rates compared to university A. If we decrease the expenditure of university B, as shown in Panel F, the institution is punished compared to university C although both exhibit the same graduation rates.

**Table 8:** Examples with three artificial universities.

*PANEL A*											
University	*E*	%*E*	*S*	*G*	%*S*	%*G*	*G*/*S*	%*S*/%*E*	%*G*/%*E*	(*G*/*S*)/*G*%	Graduation Shift
A	100	0.33	100	100	0.33	0.33	1.00	1.00	1.00	3.00	0.00
B	100	0.33	100	100	0.33	0.33	1.00	1.00	1.00	3.00	0.00
C	100	0.33	100	100	0.33	0.33	1.00	1.00	1.00	3.00	0.00
Sum	300	1.00	300	300							
*PANEL B*											
University	*E*	%*E*	*S*	*G*	%*S*	%*G*	*G*/*S*	%*S*/%*E*	%*G*/%*E*	(*G*/*S*)/*G*%	Graduation Shift
A	200	0.50	100	100	0.33	0.33	1.00	0.67	0.67	2.00	0.00
B	100	0.25	100	100	0.33	0.33	1.00	1.33	1.33	4.00	0.00
C	100	0.25	100	100	0.33	0.33	1.00	1.33	1.33	4.00	0.00
Sum	400	1.00	300	300							
*PANEL C*											
University	*E*	%*E*	*S*	*G*	%*S*	%*G*	*G*/*S*	%*S*/%*E*	%*G*/%*E*	(*G*/*S*)/*G*%	Graduation Shift
A	100	0.33	100	100	0.33	0.40	1.00	1.00	1.20	3.00	0.20
B	100	0.33	100	100	0.33	0.40	1.00	1.00	1.20	3.00	0.20
C	100	0.33	100	50	0.33	0.20	0.50	1.00	0.60	1.50	-0.40
Sum	300	1.00	300	250							
*PANEL D*											
University	*E*	%*E*	*S*	*G*	%*S*	%*G*	*G*/*S*	%*S*/%*E*	%*G*/%*E*	(*G*/*S*)/*G*%	Graduation Shift
A	100	0.40	100	100	0.33	0.40	1.00	0.83	1.00	2.50	0.17
B	50	0.20	100	100	0.33	0.40	1.00	1.67	2.00	5.00	0.33
C	100	0.40	100	50	0.33	0.20	0.50	0.83	0.50	1.25	-0.33
Sum	250	1.00	300	250							
*PANEL E*											
University	*E*	%*E*	*S*	*G*	%*S*	%*G*	*G*/*S*	%*S*/%*E*	%*G*/%*E*	(*G*/*S*)/%*G*	Graduation Shift
A	100	0.33	100	100	0.33	0.50	1.00	1.00	1.50	3.00	0.50
B	100	0.33	100	50	0.33	0.25	0.50	1.00	0.75	1.50	-0.25
C	100	0.33	100	50	0.33	0.25	0.50	1.00	0.75	1.50	-0.25
Sum	300	1.00	300	200							
*PANEL F*											
University	*E*	%*E*	*S*	*G*	%*S*	%*G*	*G*/*S*	%*S*/%*E*	%*G*/%*E*	(*G*/*S*)/%*G*	Graduation Shift
A	100	0.40	100	100	0.33	0.50	1.00	0.83	1.25	2.50	0.42
B	50	0.20	100	50	0.33	0.25	0.50	1.67	1.25	2.50	-0.42
C	100	0.40	100	50	0.33	0.25	0.50	0.83	0.63	1.25	-0.21
Sum	250	1.00	300	200							

The examples with three artificial universities in Table 8 illustrate that the idea of Bornmann et al. [1] does not appropriately account for the effect of the input variable under ceteris paribus conditions and when the shift is negative. However, in most practical applications of the shift, the limitations described in this section will not affect the efficiency results.

## Discussion

In research into higher education, the comparison of the numbers of first semester students and graduates has attracted a steady stream of interest for decades. The negative side of graduation is certainly dropout, which should be as low as possible for universities. To minimize dropout rates at universities, governments and university administrations are interested in the causes of student dropout and the subsequent career developments of these students. For example, the German Centre for Higher Education Research and Science Studies (DZHW GmbH; formerly HIS GmbH) has published several studies on the causes and motives for dropout, in addition to attrition and dropout rates at universities. In most of the studies on completion and dropout, only quotes of both phenomena have been calculated (an overview of the literature can be found in European Commission [29]). Based on the results of our study, we propose to consider also the available budget of the universities as an input variable and to calculate the ability of universities to graduate their students–in view of the available budget.

Using a comprehensive sample of 81 institutions within the period of 2008 to 2013, we show that some German universities are better able to guide students to graduation than others–given their budget constraints. We introduce the graduation shift in this study, which can be used to assess the efficiency of students’ completion success for a set of universities.

We find that the graduation shift is closely related to graduation rates. However, the graduation shift is certainly preferable, because it takes the budget of institutions into account. Although the correlation between graduation shift and rate is high, we demonstrate that there are various universities with larger differences between both indicators. The maximum difference between both indicators is 13 positions for the University of Applied Science in Landshut if the universities are ranked with respect to both indicators.

The graduation shift leads to similar ranking positions of the universities as the DEA. Since the DEA is an established instrument in efficiency measurement, the relatively high correlations could be interpreted as a validation of our new approach. However, the correlation coefficients are not perfect, which can be interpreted as follows: (1) The graduation shift does not measure efficiency in the same way as the DEA does. (2) The graduation shift can be seen as an alternative method of efficiency measurement to the DEA. (3) Some examples with three artificial universities reveal that the graduation shift is not without issues–as nearly all other indicators.

Taken as a whole, it is an advantage of the graduation shift that the differences between institutions are controlled with respect to institutional data and heterogeneity. This control is not considered in the DEA approach. It is a further advantage of the graduation shift that the computation is easy and the results are understandable to non-economists (which is not always the case with the DEA). However, when applying the graduation shift, it is worth bearing in mind that the shift has its limitations.

In this study, we used a dataset with universities of applied sciences to exemplify the calculation of the graduation shift. Future studies could elaborate this idea by computing the shift not only for universities in Germany, but also for universities in other countries. The topics of study completion and student dropout rates concern all nations with higher education systems. The results of these studies are of interest to a wide audience including students, university administrations, and policy makers.
